# The Effect of Exogenous Spermidine Concentration on Polyamine Metabolism and Salt Tolerance in Zoysiagrass (*Zoysia japonica* Steud) Subjected to Short-Term Salinity Stress

**DOI:** 10.3389/fpls.2016.01221

**Published:** 2016-08-17

**Authors:** Shucheng Li, Han Jin, Qiang Zhang

**Affiliations:** Department of Grassland Science, College of Animal Science and Technology, China Agricultural UniversityBeijing, China

**Keywords:** Zoysiagrass, polyamine metabolism, salinity stress, exogenous spermidine, antioxidant enzyme

## Abstract

Salt stress, particularly short-term salt stress, is among the most serious abiotic factors limiting plant survival and growth in China. It has been established that exogenous spermidine (Spd) stimulates plant tolerance to salt stress. The present study utilized two zoysiagrass cultivars commonly grown in China that exhibit either sensitive (cv. Z081) or tolerant (cv. Z057) adaptation capacity to salt stress. The two cultivars were subjected to 200 mM salt stress and treated with different exogenous Spd concentrations for 8 days. Polyamine [diamine putrescine (Put), tetraamine spermine (Spm), and Spd], H_2_O_2_ and malondialdehyde (MDA) contents and polyamine metabolic (ADC, ODC, SAMDC, PAO, and DAO) and antioxidant (superoxide dismutase, catalase, and peroxidase) enzyme activities were measured. The results showed that salt stress induced increases in Spd and Spm contents and ornithine decarboxylase (ODC), S-adenosylmethionine decarboxylase (SAMDC), and diamine oxidase (DAO) activities in both cultivars. Exogenous Spd application did not alter polyamine contents via regulation of polyamine-degrading enzymes, and an increase in polyamine biosynthetic enzyme levels was observed during the experiment. Increasing the concentration of exogenous Spd resulted in a tendency of the Spd and Spm contents and ODC, SAMDC, DAO, and antioxidant enzyme activities to first increase and then decrease in both cultivars. H_2_O_2_ and MDA levels significantly decreased in both cultivars treated with Spd. Additionally, in both cultivars, positive correlations between polyamine biosynthetic enzymes (ADC, SAMDC), DAO, and antioxidant enzymes (SOD, POD, CAT), but negative correlations with H_2_O_2_ and MDA levels, and the Spd + Spm content were observed with an increase in the concentration of exogenous Spd.

## Introduction

Due to the generation of a hyperosmotic effect by reducing the soil water potential, salt stress, particularly short-term salt stress, is one of the most serious abiotic factors limiting productivity in turf grass (Alshammary et al., 2004; Ahn et al., 2015). Furthermore, salt stress induces a hypertonic effect, and these ions are directly toxic to plant metabolism and nutrition. Additionally, free radicals induce structural damage in plant cells, causing a loss of turgidity and thereby weakening the organism. However, plants have evolved various defense mechanisms to mitigate salt damage, including accumulation of osmolytes such as sugars, glycine betaine, and proline and adaptations to salt stress such as Na^+^/H^+^ antiporters (Qi et al., 2014). Salinity stress also increases the levels of polyamines (Chattopadhayay et al., 2002).

**Figure 1:**
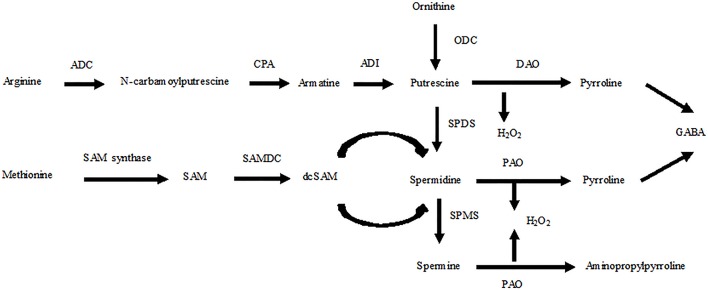
**Schematic presentation of the PA biosynthetic pathways for Put, Spd, and Spm in plants and relationships with ethylene biosynthesis**. ADC, arginine decarboxylase; CPA, N-carbamoylputrescine amidohydrolase; ADI, agmatine deaminase; DAO, diamine oxidase; PAO, polyamine oxidase; SAMDC, S-adenosylmethionine decarboxylase; dc-SAM, decarboxylated S-adenosylmethionine; SPDS, spermidine synthase; SPMS, spermine synthase; GABA, c-aminobutyric acid.

Polyamines (PAs) are ubiquitous compounds in plant cells that are essential for growth and development. PAs also play an important role in the response of plants to adverse environmental conditions due to their polycationic nature (Puyang et al., 2015; Pál et al., 2015). These compounds mainly exist in three forms in plant cells, diamine putrescine (Put), triamine spermidine (Spd), and tetraamine spermine (Spm), each of which may be present in a free, soluble conjugated or insoluble bound form. Soluble conjugated forms, such as phenolic compounds, are covalently conjugated to small molecules, whereas insoluble bound forms are covalently bound to macromolecules, such as nucleic acids and proteins (Gill and Tuteja, 2010). In plants, arginine decarboxylase (ADC), ornithine decarboxylase (ODC), and S-adenosylmethionine decarboxylase (SAMDC) are the key enzymes responsible for synthesizing PAs. Arginase and ODC convert ornithine to Put, which is also synthesized via agmatine through three sequential reactions catalyzed by ADC, agmatine iminohydrolase (AIH), and N-carbamoylputrescine amidohydrolase (CPA). Spd and Spm are produced from Put in plants through successive addition of aminopropyl groups from decarboxylated S-adenosylmethionine (dc-SAM), which is generated from SAM by SAMDC. Conversely, PAs are degraded by diamine oxidase (DAO) and polyamine oxidase (PAO; Kusano et al., 2008; Tavladoraki et al., 2012; Figure 1).

Much evidence to date has shown that exogenous application of PAs (di- and tri- and tetra-amines) enhances tolerance to salinity stress in plants by stabilizing membrane and cellular structures, scavenging free radicals, modulating ion channels, maintaining the cation-anion balance, and energizing cells via stimulation of ATP synthesis (Hartung et al., 2002; Nuttall et al., 2003; Shi and Sheng, 2005; Yang et al., 2007). Indeed, application of exogenous PAs can effectively augment plant salinity tolerance and ultimately improve plant productivity under high-salinity conditions (Ndayiragije and Lutts, 2006), and studies in various species have indicated that such enhance salinity tolerance occurs through the synergy of a number of mechanisms (Ndayiragije and Lutts, 2006; Roychoudhury et al., 2011; Saleethong et al., 2011; Velarde-Buendía et al., 2012; Shu et al., 2013; Zhang et al., 2014). Among the three major PAs, Spd is most closely associated with stress tolerance in plants (Shen et al., 2000).

To date, there have been substantial efforts toward improving salinity tolerance in plants through transgenic techniques, several of which have been widely applied in *Arabidopsis thaliana* and transgenic rice overexpressing genes for PA biosynthetic enzymes (Roy and Wu, 2001; Kasukabe et al., 2004, 2006). In addition, exogenous application of Spd dramatically reversed the observed cinnamic acid (CA)-induced effects on Spd + Spm and partially restored the PA ratio and RuBPC activity in leaves, whereas methylglyoxial-bis, an inhibitor of SAMDC, exacerbated the negative effects induced by CA (Huang and Bie, 2010). Exogenous application of Spd was also found to affect systemic glucosylsalicylic acid levels and ADC gene expression in tobacco leaves (Lazzarato et al., 2009). It has been shown that exogenous Spd alters the activities of polyamine degradation enzymes (PAO, DAO) in many species under salt stress (Hu et al., 2014).

Previous studies have indicated that exogenous Spd application reverses the increases in MDA content and electrolyte leakage caused by chilling. Moreover, high endogenous free PA contents have been observed (Zhang et al., 2009). Shoeb et al. (2001) reported that modulation of cellular PA levels and the Put:Spd ratio by exogenous PA (Put, Spd) application or treatment with difluoromethylarginine, a PA biosynthesis inhibitor, led to plant regeneration in poorly responding genotypes. Li et al. (2015) also reported that exogenous Spd confers short-term salinity tolerance in creeping bentgrass, likely by inducing antioxidant enzymes and affecting the activities of enzymes involved in PA metabolism. Some authors have observed that exogenous application of 0.1 mM Spd affects PA metabolism (Duan et al., 2008), and others have observed that 0.20 or 0.25 mM exogenous Spd has similar effects on PA metabolism in plants subjected to salt stress (Li et al., 2015). Such differences are related to the concentration of exogenous Spd applied and the cultivars examined (Zrig et al., 2011).

The objective of this study was to understand the effect of exogenous Spd concentrations on PA metabolism and the physiological and biochemical responses involved in salinity tolerance, especially their correlations, in a salinity-sensitive cultivar (cv. Z081) and a salinity-tolerant cultivar (cv. Z057). Parameters such as the contents of PAs (Put, Spd, and Spm) and activities of PA biosynthetic enzymes (ADC, ODC, SAMDC, PAO, and DAO) and antioxidant enzymes (SOD, POD, and CAT), as well as the degree of lipid peroxidation, were measured. We hypothesized that different concentrations of exogenous Spd would contribute to salt tolerance in both cultivars. Our second hypothesis was that correlations between Spd + Spm contents and H_2_O_2_ and MDA accumulation exist with changes in the concentration of exogenous Spd. Our third hypothesis was that correlations also exist between PA metabolism, PA biosynthetic enzymes and antioxidant enzymes with increases in exogenous Spd in the two cultivars analyzed.

## Materials and methods

### Chemicals

NaOH (AR, > 96%), HCl (AR, 36%), K_2_HPO_4_ (AR, ≥ 99.0%), KH_2_PO_4_ (AR, ≥ 99.5%), NaCl (AR, ≥ 99.5%), benzoyl chloride (AR, 99%), perchloric acid (AR, 99%), 1, 6-hexanediamine (AR, ≥ 99.8%), pyridoxal phosphate (AR, 98%), EDTA (AR, ≥ 99.5%), phenylmethylsulfonyl fluoride (GC, > 98%), ascorbic acid (AR, ≥ 99.7%), polyvinylpyrrolidone (AR, > 95%), L-ornithine (BR, 99%), L-arginine (BR, ≥ 99.0%), S-adenosyl methionine (BR, ≥ 98.0%), 4-aminoantipyrine/N,N-dimethylaniline (AR, 99%), horseradish peroxidase (250 units ml^−1^), acetone (AR, 99.9%), H_2_SO_4_ (AR, 98%), thiobarbituric acid (AR, ≥ 99%), trichloroacetic acid (AR, ≥ 99%), nitroblue tetrazolium (AR, 98%), methionine (BR, 99.0%), riboflavin (BR, 99%), guaiacol (GC, > 99%), spermidine (GC, > 99%), putrescine (CP, 99%), spermine (AR, ≥ 97%).

### Plant material and treatments

Two zoysiagrass (*Zoysia japonica* Steud) cultivars, “Z081” and “Z057,” were used in this study (Table 1). Z081 is sensitive to saline conditions, whereas Z057 is tolerant to saline conditions (Li et al., 2012). The plants were cultivated in tanks containing 1/2 Hoagland solution (pH 6.6 ± 0.1, EC 1.8–2.0 dsm^−1^) in the greenhouse of China Agricultural University throughout the year and mowed once a week to maintain a height of 10 cm. Zoysiagrass plants of consistent size were transplanted into tanks containing 20 l of 1/2 Hoagland solution (pH 6.6 ± 0.1, EC 1.8–2.0 dsm^−1^). The roots were clipped to a length of 5 cm before initiating the six treatments: (1) control—1/2 Hoagland solution alone; (2) salt stress control—1/2 Hoagland solution + 200 mM NaCl; (3) 1/2 Hoagland solution + 200 mM NaCl + 0.15 mM Spd; (4) 1/2 Hoagland solution + 200 mM NaCl + 0.30 mM Spd; (5) 1/2 Hoagland solution + 200 mM NaCl + 0.45 mM Spd; (6) 1/2 Hoagland solution + 200 mM NaCl + 0.60 mM Spd. The NaCl concentration was gradually increased in 50 mM increments every day to avoid salinity shock. Spd (Sigma Chemical Co., St. Louis, MO, USA) was added after the salt level reached 200 mM. The solutions were constantly aerated using pumps. The day and night air temperatures were 25–28°C and 17–20°C, respectively, and the relative humidity of the greenhouse was 60–70% (China Agricultural University, Haidian, Beijing, China).

**Table 1:** *****Zoysia japonica*** cultivars used in the study, the growth conditions and the source of the plants**.

**Cultivar**	**Salinity tolerance**	**Species**	**Source sponsor**	**Source location**
Z081	150 mM	*Z. japonica*	Qingdao, Shandong	36°05′N, 120°20′E
Z057	340 mM	*Z. japonica*	HuaguoShan, Lianyungang	34°36′N, 119°12′E

Root samples were collected in triplicate 8 days after initiation of the salinity treatments.

### Determination of root growth

Root length was measured using a ruler, and dry weight was assessed by weighing roots after drying at 75°C in an oven for 72 h. The relative water content (RWC) was calculated according to the following formula:

RWC = (fresh weight − dry weight)/(saturation weight − dry weight)

### Polyamine analysis

#### Standard sample and standard curve

First, 2 ml 2 N NaOH and 15 μl benzoyl chloride were added to 100 μl 1 mM Put, Spd, and Spm standards. The samples were then vortexed vigorously and incubated for 30 min at 37°C. Next, a 4 ml saturated NaCl solution was added; 1.5 ml of the ether phase was dried and redissolved in 1 ml methanol.

Put, Spd, and Spm standards were prepared in 1 mM benzoylated solution, and a standard curve was generated using standards of different densities (0.03, 0.06, 0.12, 0.15, 0.25, 0.50, 0.75, 1.0 nmol).

#### Polyamine analysis

PAs were extracted according to the methods of Sharma and Rajam (1995), with some modifications. Fresh root samples (0.3 g) were homogenized in cold perchloric acid (PCA, 4 ml, 5% v/v), followed by incubation at 4°C for 1 h. Next, 1, 6-hexanediamine was added to the homogenate as an internal standard, and the mixture was centrifuged at 12,000 × g for 30 min at 4°C. The supernatant was subsequently used for determination of free and soluble conjugated PAs, and the pellet was used for determination of insoluble bound PAs. To obtain soluble conjugated PAs, 1 ml PCA extract was blended with 5 ml 6 N HCl and hydrolyzed at 110°C for 18 h in flame-sealed glass ampules. The HCl was evaporated by heating at 70°C, and the residue was suspended in 2 ml 5% PCA after acid hydrolysis, followed by centrifugation at 12,000 × g for 30 min at 4°C. The acid-soluble polyamine solution contained free PAs and conjugates liberated from PAs. To obtain insoluble bound PAs, the pellet was rinsed four times with 5% PCA to remove any trace of soluble PAs and resuspended in 5 ml 6 N HCl. This solution was hydrolyzed using the same procedure as described above.

PAs were recovered from the pellet, and the hydrolyzed supernatant and non-hydrolyzed supernatant were benzoylated as follows. An aliquot of the supernatant containing 2 ml 2 N NaOH and 15 μl benzoyl chloride was vortexed vigorously and then incubated for 30 min at 37°C. Next, 4 ml saturated NaCl solution was added, and 1.5 ml of the ether phase was dried and redissolved in 1 ml methanol (60% w/v). The solution was stored at −20°C under air-tight conditions.

PAs were assayed via high-performance liquid chromatography (HPLC). A 10 μl aliquot of a methanol solution of benzoyl polyamines was injected into a 20 ml loop and loaded onto a 5 μm particle size C18 reverse-phase, 4.6 × 250 mm column (Eka Chemicals, Bohus, Sweden). The temperature of the column was maintained at 25°C. The samples were eluted with 64% methanol at a flow rate of 0.8 ml min^−1^ that was maintained by a Dionex P680 Pump. The PA peaks were detected with a UV detector at 254 nm. The concentrations of soluble conjugated forms were calculated by subtracting the free PA concentration from acid-soluble PA concentration.

### Analysis of polyamine biosynthetic enzyme activity

Fresh root samples (0.3 g) were homogenized in 100 mM potassium phosphate buffer (pH 8.0) containing 0.1 mM phenylmethylsulfonyl fluoride, 1 mM pyridoxal phosphate (PLP), 5 mM EDTA, 25 mM ascorbic acid, and 0.1% polyvinylpyrrolidone. The solution was then centrifuged at 12,000 × g for 40 min at 4°C. The supernatant was dialyzed at 4°C against 3 ml 100 mM potassium phosphate buffer (pH 8.0) containing 0.05 mM PLP, 0.1 mM DTT, and 0.1 mM EDTA in darkness for 24 h. The dialyzed extract was used for enzyme assays.

Enzyme activity was determined according to a previously described procedure (Matsuda, 1984), with some modifications. The activities of ODC, ADC, and SAMDC were measured using reaction mixtures prepared with 0.3 ml of the dialyzed enzyme extract and 100 mM Tris-HCl buffer (pH 8.0), 50 μM pyridoxal phosphate, 5 mM EDTA, and 5 mM DTT. The reactions were incubated at 37°C for 2 min, and 0.2 ml 25 mM L-ornithine, 0.2 ml 25 mM L-arginine (pH 7.5) or 0.2 ml 25 mM SAM was then added. The reaction mixtures were incubated at 37°C for 30 min, and PCA was added to a final concentration of 5%. The reaction mixtures were centrifuged at 3000 × g for 10 min, and the supernatants (0.5 ml) were mixed with 1 ml 2 mM NaOH and 10 μl benzoyl chloride. The mixture was stirred for 20 s, and 2 ml NaCl solution and 3 ml ether were added and incubated at 37°C for 30 min while stirring thoroughly. The reaction was then centrifuged at 1500 × g for 5 min and extracted with 3.0 ml ether; the ether phase (1.5 ml) was evaporated to dryness and redissolved in 3 ml 60% methyl alcohol. Finally, the solution was subjected to UV light at a wavelength of 254 nm.

### Assay for diamine and polyamine oxidase activities

The activities of DAO and PAO were determined by measuring the generation of H_2_O_2_, a product of PA oxidation, as described previously (Su et al., 2005), with some modifications. Fresh samples were homogenized in 100 mM potassium phosphate buffer (pH 6.5), and the homogenate was centrifuged at 10,000 × g for 20 min at 4°C. The supernatant was used for the enzyme assay. The reaction mixtures contained 25 ml potassium phosphate buffer (100 mM, pH 6.5), 0.2 ml 4-aminoantipyrine/N,N-dimethylaniline reaction solution, 0.1 ml horseradish peroxidase (250 units ml^−1^), and 0.2 ml of the enzyme extract. The reactions were initiated by adding 15 μl 20 mM Put to determine DAO activity or 20 mM Spd + Spm to determine PAO activity. One unit of enzyme activity was defined as a change in absorbance of 0.001 units.

### Evaluation of free radical production

Fresh root samples (0.3 g) were homogenized in cold acetone (5 ml) and centrifuged at 10000 × g for 15 min at 4°C. The supernatant was added to concentrated hydrochloric acid solution containing 20% TiCl_4_ (0.1 ml) and concentrated ammonia (0.2 ml). The reaction mixture was centrifuged for 10 min at 8000 × g and 4°C after a 5 min reaction at 25°C. After washing twice with cold acetone, 3 ml 1 M H_2_SO_4_ was added to the pellet. We measured absorption at 410 nm, and the H_2_O_2_ concentration was calculated using a standard curve.

### Lipid peroxidation assay

The malondialdehyde (MDA) content was determined using the thiobarbituric acid (TBA) method (Dhindsa et al., 1981). Fresh root samples (0.3 g) were homogenized in 5 ml of 5% trichloroacetic acid (TCA) centrifuged at 15,000 × g for 20 min. A 0.5 ml aliquot of the supernatant was add to 1 ml 20% (w/v) TCA containing 0.5% (w/v) TBA. The reaction was placed in boiling water for 30 min, quickly cooled and centrifuged at 10,000 × g for 10 min. Absorbance measurements were obtained at 532 and 600 nm.

### Antioxidant enzyme activities

Fresh root samples (0.1 g) were homogenized with 1 ml ice-cold phosphate buffer (50 mM, pH 7.8) containing 1 mM EDTA and 4% PVP. The homogenate was centrifuged at 10,000 rpm at 4°C for 15 min, and the supernatant was used for determining the activities of POD, SOD, and CAT at 4°C.

Superoxide dismutase (SOD, EC1.15.1.1) activity was determined by observing the inhibition of NBT reduction. The 3 ml reaction mixture contained phosphate buffer (50 mM, pH 7.8), EDTA (0.1 mM), methionine (130 mM), NBT (0.75 mM), riboflavin (0.02 mM), and enzyme extract (0.1 mM), with the riboflavin added last. The reaction mixture was illuminated for 15 min. The calibration standards consisted of non-illuminated and illuminated reactions without supernatant. One unit of activity was defined as the amount of enzyme causing 50% inhibition of the reduction of nitroblue tetrazolium chloride, as assessed at 560 nm.

Peroxidase (POD, EC 1.11.1.7) activity was determined using guaiacol. The reaction mixture contained guaiacol solution (0.02 ml), hydrogen peroxide solution (0.01 ml), phosphate buffer (3 ml, pH 7.0), and enzyme extract (0.02 ml). The reaction was initiated by addition of the enzyme extract. One unit of activity was equal to an increase in 1 absorbance unit per minute at 470 nm.

Catalase (CAT, EC 1.11.1.6) activity was determined by monitoring the initial H_2_O_2_ disappearance rate. Phosphate buffer (50 mM, pH 7.0), H_2_O_2_ (20 mM), and enzyme extract (0.1 ml) were added to the reaction solution (3 ml). The reaction was initiated by addition of the enzyme extract. The reduction of H_2_O_2_ was observed for at least 3 min at 240 nm.

### Statistical analysis

Root growth measurements were replicated 40 times. Each experimental treatment was completely random and was designed to be replicated at least three times. The results are expressed as the mean ± standard errors (SE). One-way analysis of variance (ANOVA) with an LSD test was used to determine the significance of the observed differences between treatments.

## Results

### Plant growth

Biometric analysis indicated that 8 days of salt treatment significantly reduced root growth in both cultivars (*p* < 0.05). Under salt stress, the fresh root weight, root length, and relative root water content increased initially but later declined in both cultivars as the Spd concentration increased (Table 2). To some extent, exogenous Spd alleviated the salinity-induced reduction in growth, with a greater effect in cv. Z081 than in cv. Z057. For example, compared with untreated plants grown under salt stress, 0.3 mM Spd enhanced the fresh root weight, root length, and relative root water content of cv. Z081 and cv. Z057 by 26, 13, and 16% and 18.7, 12.6, and 13.2%, respectively. However, a high concentration of Spd repressed the growth of cv. Z081 under salt stress.

**Table 2:** **Effects of the addition of different concentrations of exogenous spermidine on the growth and water content of zoysiagrass roots exposed to 200 mM NaCl for 8 days**.

**Cultivar**	**Treatment**	**Root fresh weight (g /cm^2^)**	**Root length (cm)**	**Root relative water content (%)**
Z081	Control	0.395 ± 0.03a	6.67 ± 0.06a	93.1 ± 3.7a
	NaCl	0.271 ± 0.04d	5.49 ± 0.08e	74.2 ± 1.4d
	NaCl + 0.15 mM Spd	0.339 ± 0.02b	6.21 ± 0.07b	86.4 ± 2.3b
	NaCl + 0.3 mM Spd	0.341 ± 0.04b	6.19 ± 0.08b	86.2 ± 2.4b
	NaCl + 0.45 mM Spd	0.326 ± 0.01c	5.94 ± 0.10c	81.3 ± 1.2c
	NaCl + 0.6 mM Spd	0.241 ± 0.04e	5.27 ± 0.09e	71.5 ± 2.7e
Z057	Control	0.423 ± 0.04a	6.99 ± 0.04a	93.9 ± 2.9a
	NaCl	0.331 ± 0.03d	5.97 ± 0.06e	78.3 ± 2.2d
	NaCl + 0.15 mM Spd	0.357 ± 0.02c	6.51 ± 0.06c	88.5 ± 1.9b
	NaCl + 0.3 mM Spd	0.383 ± 0.02b	6.69 ± 0.02b	88.6 ± 2.1b
	NaCl + 0.45 mM Spd	0.361 ± 0.02c	6.54 ± 0.09c	84.2 ± 1.5c
	NaCl + 0.6 mM Spd	0.332 ± 0.03d	6.27 ± 0.08d	79.0 ± 2.4d

*Data represent the mean ± SE of three independent experiments. Values in the table sharing the same letters are not significantly different (p < 0.05; Duncan's multiple range test)*.

### Polyamine levels

**Figure 2:**
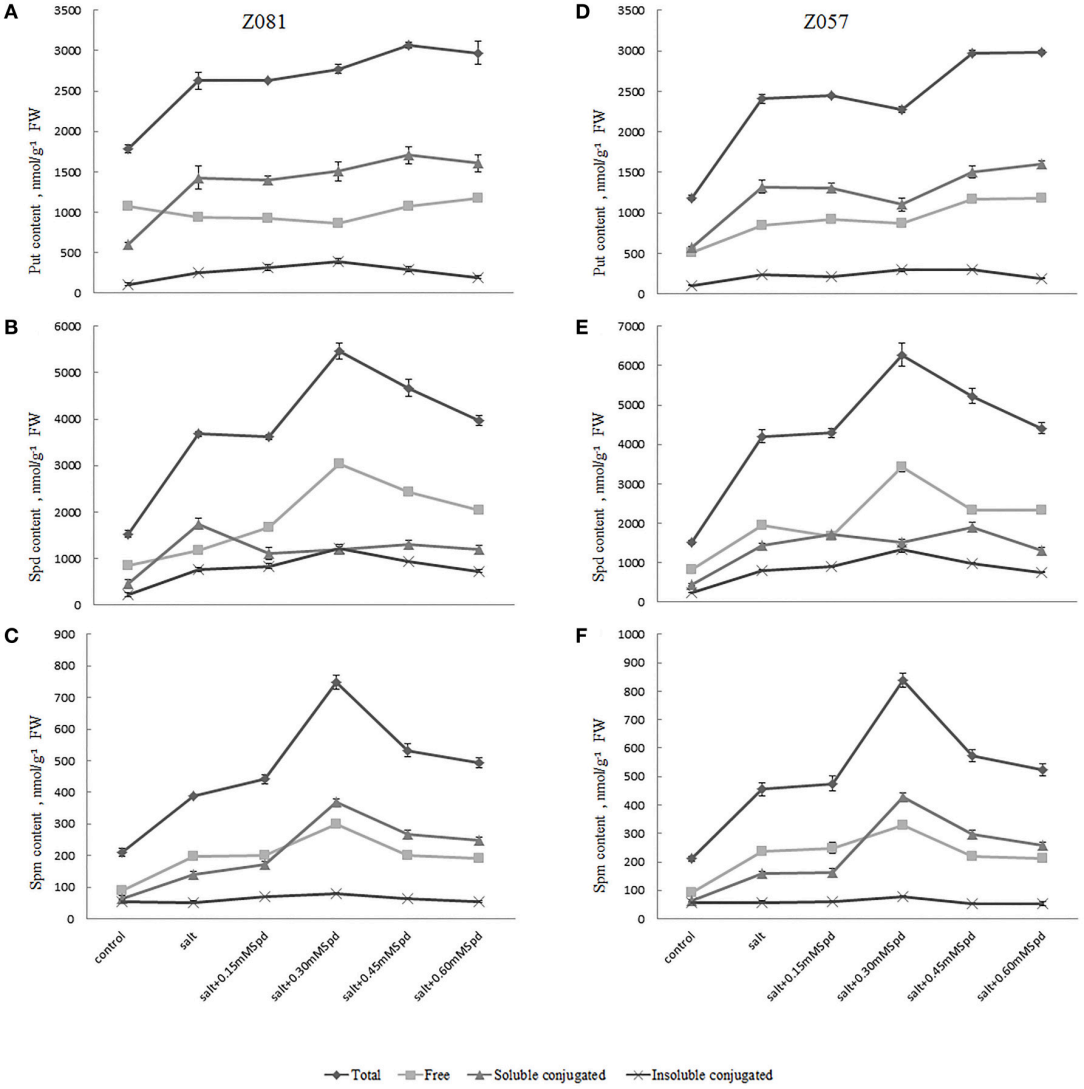
**Effects of exogenous spermidine (0, 0.15, 0.30, 0.45, 0.60 mM) on PA contents in the roots of cv. Z081 (A–C) and cv. Z057 (D–F) grown under 200 mM NaCl stress**. **(A,D)** Put (total, free, soluble conjugated, insoluble conjugated) content; **(B,E)** Spd (total, free, soluble conjugated, insoluble conjugated) content; **(C,F)** Spm (total, free, soluble conjugated, insoluble conjugated) content. Data represent the means ± SE of three replicates. Values in a single column sharing the same letters are not significantly different (*p* < 0.05) (Duncan’s multiple range test).

The biosynthetic pathways of the major PAs Put, Spd, and Spm are shown in Figure 2. These three main PAs differ in the positive changes observed in the physiology of treated cells. In previous reports, Spd and Spm levels and the (Spd + Spm)/Put ratio increased with salinity in all species showing increased salinity tolerance (Figure 2, Table 3). In the present study, we measured PA (Put, Spd, and Spm) levels (Figure 2) and the ratio of (Spd + Spm)/Put in both cultivars (Table 3). Salt stress increased the total PA (Put, Spd, and Spm) content in both cultivars (Figure 2); however, little change in the Spd and Spm contents in the roots of cv. Z081 was observed under salinity stress. In general, the PA contents of both cultivars first increased and then declined with increasing concentrations of exogenous Spd, whereas the contents, except for Put, increased dramatically and then peaked under 0.3 mM Spd treatment (Figure 2).

**Table 3:** **Changes in polyamine content in zoysiagrass under salt stress**.

**Endogenous polyamines content (nmol/g^−1^ fw)**				
**Cultivar**	**Treatment**	**Put**	**Spd** + **Spm**	**(spd** + **spm)/Put**
Z081	Control	1781 ± 49d	1744 ± 83d	0.98 ± 0.03c
	NaCl	2626 ± 101c	4070 ± 60c	1.55 ± 0.06b
	NaCl + 0.15 mM Spd	2631 ± 17c	4066 ± 41c	1.55 ± 0.03b
	NaCl + 0.3 mM Spd	2772 ± 56bc	6220 ± 198a	2.24 ± 0.12a
	NaCl + 0.45 mM Spd	3072 ± 39a	5204 ± 201b	1.69 ± 0.09b
	NaCl + 0.6 mM Spd	2972 ± 142ab	4466 ± 164c	1.50 ± 0.02b
Z057	Control	1186 ± 30d	1715 ± 57d	1.45 ± 0.01c
	NaCl	2407 ± 51b	4664 ± 188c	1.94 ± 0.05b
	NaCl + 0.15 mM Spd	2444 ± 21b	4762 ± 146c	1.95 ± 0.08b
	NaCl + 0.3 mM Spd	2772 ± 36c	7112 ± 317a	3.1 ± 0.11a
	NaCl + 0.45 mM Spd	2976 ± 28a	5803 ± 205b	1.95 ± 0.08b
	NaCl + 0.6 mM Spd	2982 ± 21a	4933 ± 167c	1.65 ± 0.06c

*The data represent the mean values ± standard error (SE) from at least three independent experiments. Means with different letters are significantly different at p < 0.05*.

Salt stress also caused an increase in the three forms (free form, soluble conjugated form, and insoluble bound form) of Put, Spd, and Spm. The levels of Put (insoluble bound), Spd (free, insoluble bound), and Spm (free, soluble conjugated, insoluble bound) initially increased and then declined with increasing concentrations of exogenous Spd in cv. Z081 (Figures 2A–C). Similar results were observed in cv. Z057, except for free and soluble conjugated Put (Figures 2D–F). Conversely, the levels of free Put first decreased and then rose slightly in the two cultivars with increasing exogenous Spd concentrations.

Additionally, the ratio of (Spd + Spm)/Put and the Spd + Spm contents, which are related to increased tolerance to salt stress, first increased and then decreased, peaking in both cultivars with 0.3 mM exogenous Spd (Table 3).

### Polyamine biosynthetic enzyme activities

**Figure 3:**
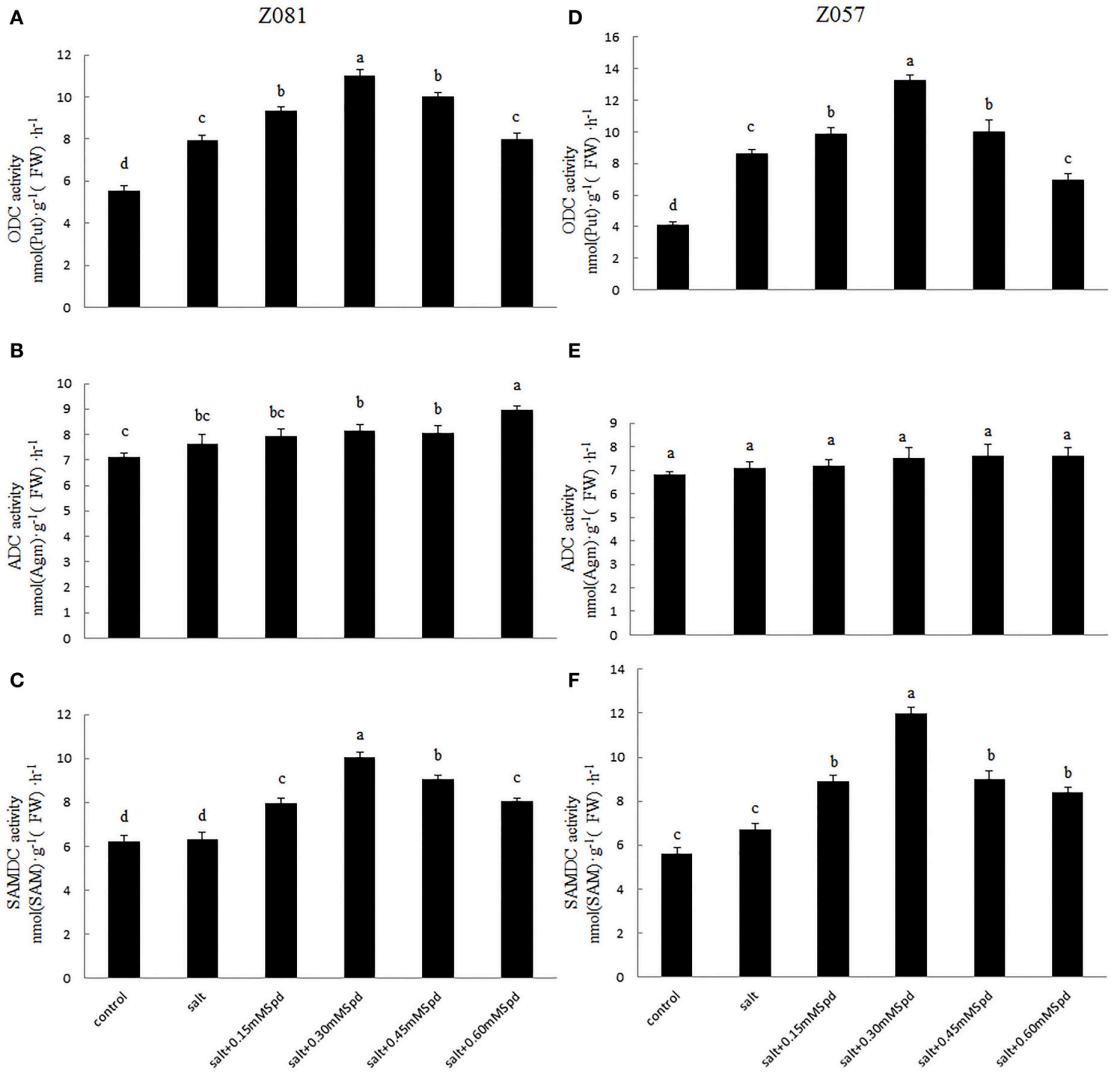
**Effects of exogenous spermidine (0, 0.15, 0.30, 0.45, 0.60 mM) on activities of ODC, ADC, and SAMDC in the roots of cv. Z081 (A–C) and cv. Z057 (D–F) grown under 200 mM NaCl stress**. **(A,D)** ODC activity; **(B,E)** ADC activity; **(C,F)** SAMDC activity. Data represent the means ± SE of three replicates. Values in a single column sharing the same letters are not significantly different (*p* < 0.05) (Duncan’s multiple range test).

The activities of several representative enzymes, including ODC and ADC (Figure 1), were measured in roots to determine the effects of exogenous Spd application on PA synthesis under salt stress. As shown in Figures 3A,D, ODC activity in the roots of both cultivars was enhanced under salt stress. The exogenous Spd-induced increase in ODC activity was greater in cv. Z057 than in cv. Z081, and the enhancement first increased and then decreased with increasing concentration of exogenous Spd. However, compared with the control roots under salt stress, ODC activity was inhibited in cv. Z081 by treatment with 0.6 mM Spd. In contrast, ADC activity did not respond to salt stress, and exogenous Spd had no significant effect on ADC activity in either cultivar (Figures 3B,E).

The activity of SAMDC increased slightly when the roots were exposed to salt stress. As the concentration of exogenously added Spd increased, SAMDC activity was first augmented and then diminished. Spd treatment at 0.3 mM resulted in the greatest SAMDC activity in both cultivars, with a greater effect in cv. Z057 than in cv. Z081 (Figures 3C,F).

### Polyamine-degrading enzyme activities

**Figure 4:**
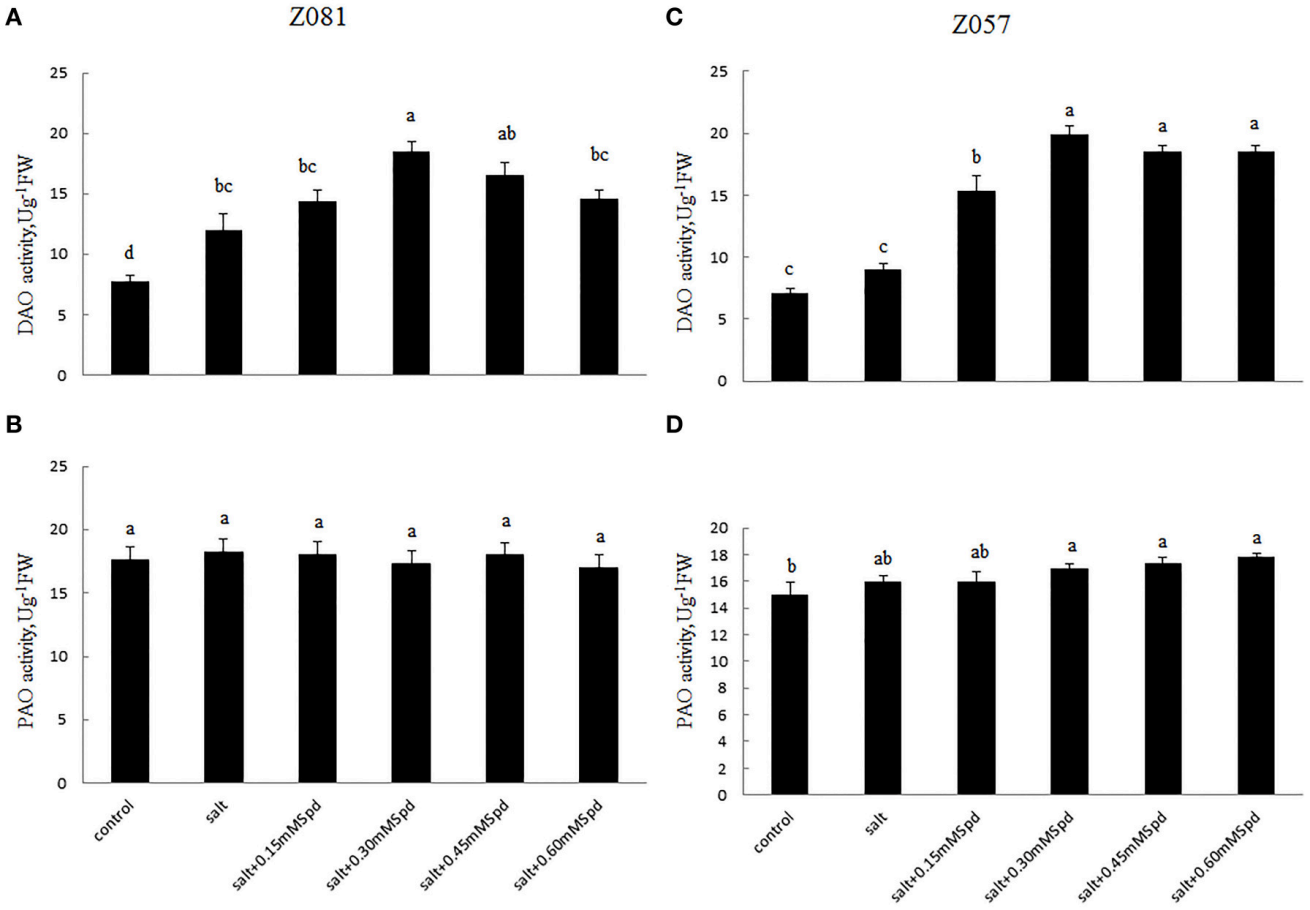
**Effects of exogenous spermidine (0, 0.15, 0.30, 0.45, 0.60 mM) on the activities of DAO and PAO in the roots of cv. Z081 (A,B) and cv. Z057 (C,D) grown under 200 mM NaCl stress**. **(A,C)** DAO activity; **(B,D)** PAO activity. Data represent the means ± SE of three replicates. Values in a single column sharing the same letters are not significantly different (*p* < 0.05) (Duncan’s multiple range test).

To elucidate the polyamine metabolism underlying the salt tolerance induced by exogenous Spd, we measured two PA-degrading enzymes. The activity of DAO in roots increased rapidly in both cultivars under salinity stress (Figures 4A,C). The DAO activity in roots treated with exogenous Spd initially increased and then decreased in a dose-dependent manner in both cultivars. With increasing exogenous Spd, DAO activity showed a faster downward trend in Z081 than in Z057 (Figures 4A,C).

Salinity stress only induced a slight increase in PAO activity. Moreover, compared with the corresponding control, Spd application did not have a significant effect on root PAO activity in either cultivar (Figures 4B,D).

### H_2_O_2_ concentration and lipid peroxidation

**Figure 5:**
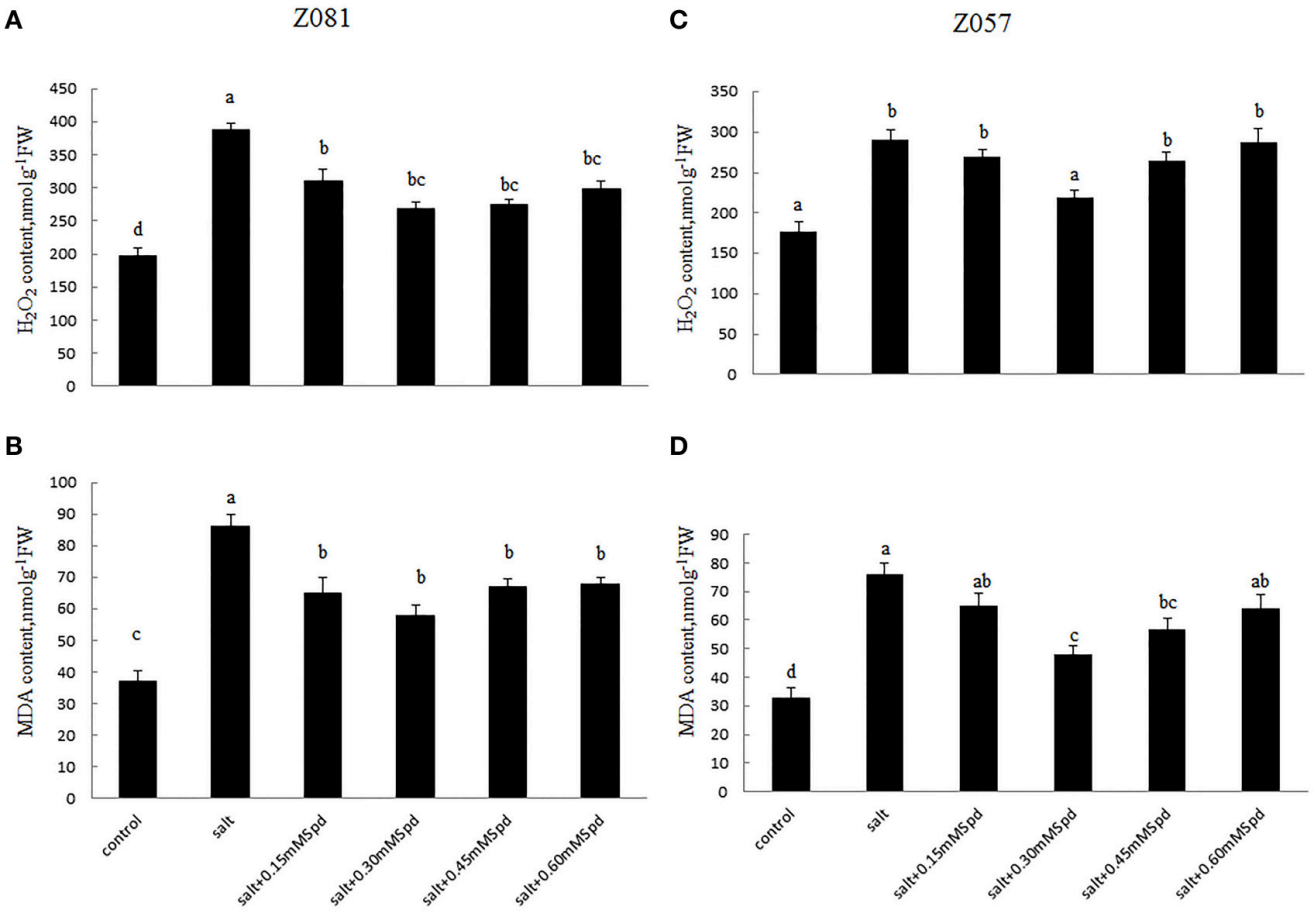
**Effects of exogenous spermidine (0, 0.15, 0.30, 0.45, 0.60 mM) on the levels of H_2_O_2_ and MDA in the roots of cv. Z081 (A,B) and cv. Z057 (C,D) grown under 200 mM NaCl stress**. **(A,C)** H_2_O_2_ content; **(B,D)** MDA content. Data represent the means ± SE of three replicates. Values in a single column sharing the same letters are not significantly different (*p* < 0.05) (Duncan’s multiple range test).

Compared with the controls, salinity stress led to greater increases in H_2_O_2_ and MDA levels in cv. Z081 than in cv. Z057, and treatment with exogenous Spd reduced the levels of H_2_O_2_ and malondialdehyde (MDA) in both cultivars. However, this decrease was much smaller in cv. Z057 than in cv. Z081. The levels of H_2_O_2_ and MDA initially increased and then decreased, with the minimum values in both cultivars observed in the roots of plants treated with 0.3 mM Spd (Figure 5).

### Antioxidant enzyme activities

**Figure 6:**
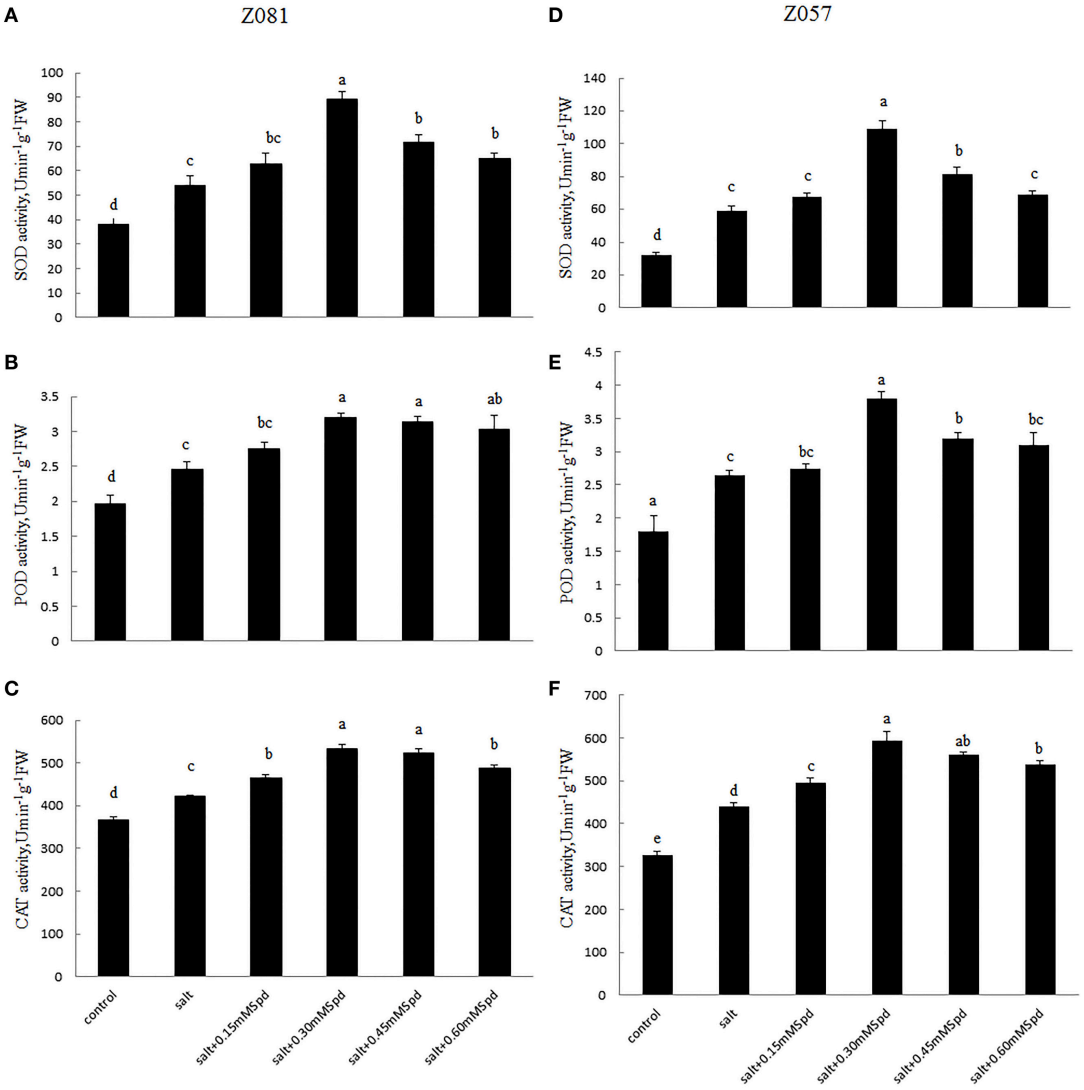
**Effects of exogenous spermidine (0, 0.15, 0.30, 0.45, 0.60 mM) on the activities of SOD, POD, and MDA in the roots of cv. Z081 (A–C) and cv. Z057 (D–F) grown under 200 mM NaCl stress**. **(A,D)** SOD activity; **(B,E)** POD activity; **(C,F)** CAT activity. Data represent the means ± SE of three replicates. Values in a single column sharing the same letters are not significantly different (*p* < 0.05) (Duncan’s multiple range test).

The activities of several representative antioxidant enzymes, including SOD, POD, and CAT, were measured in zoysiagrass to determine the physiological effect of exogenous Spd on these antioxidant enzymes within the context of salt stress. During salinity treatment (200 mM NaCl), SOD activity was determined to be 54.1 U min^−1^ g^−1^ FW but was found to be as high as 89.2 U min^−1^ g^−1^ FW in cv. Z081 treated with 0.3 mM exogenous Spd (Figure 6A). Similar results were observed in cv. Z057, and the extent of increase in the roots due to Spd application was much greater in cv. Z057 than in cv. Z081 (Figure 6D). Regarding POD and CAT activities, treatment of salinity-stressed plants with Spd resulted in a tendency of increase followed by decrease in both cultivars. For example, treatment with 0.3 mM Spd increased the activities of POD and CAT by 30.1 and 26.8% and 43.4 and 34.7% in cv. Z081 and cv. Z057, respectively, compared with treated, salinity-stressed plants (Figures 6B,C,E,F).

### Correlation analysis

The correlation coefficients between exogenous Spd level indexes, as analyzed by Pearson's correlation, are listed in Tables 4, 5. The exogenous Spd indexes related to several parameters showed significant correlations. In both cultivars, the Spd + Spm contents displayed positive correlations with PA biosynthetic enzymes and antioxidant enzymes, though the Spd + Spm contents showed a negative correlation with MDA and H_2_O_2_ levels (Tables 4, 5). DAO activity also showed a positive correlation with Spd + Spm contents in both cultivars (Tables 4, 5).

**Table 4:** **Pearson's correlation coefficients among PA contents and physiological and biochemical parameters in cv. Z081 exposed to salt stress and treated with exogenous Spd (0, 0.15, 0.30, 0.45, 0.60 mM)**.

	**Put**	**Spd + Spm**	**ADC**	**ODC**	**SAMDC**	**PAO**	**DAO**	**H_2_O_2_**	**MDA**	**SOD**	**POD**	**CAT**
Put	−	0.299	0.390	0.143	0.377	−0.089	0.316	−0.472	−0.235	0.197	0.406	0.511
Spd + Spm	0.299	−	0.233	0.851^**^	0.888^**^	0.076	0.875^**^	−0.573^*^	−0.493	0.935^**^	0.569^*^	0.746^**^
ADC	0.390	0.233	−	0.051	0.396	0.336	0.511	−0.137	−0.005	0.392	0.109	0.161
ODC	0.143	0.851^**^	0.051	−	0.891^**^	0.224	0.874^**^	−0.572^*^	−0.554^*^	0.873^**^	0.441	0.679^**^
SAMDC	0.377	0.888^**^	0.396	0.891^**^	−	0.067	0.947^**^	−0.762^**^	−0.678^**^	0.946^**^	0.666^**^	0.852^**^
PAO	−0.089	0.076	0.336	0.224	0.067	−	0.345	0.490	0.552^*^	0.151	−0.600^*^	−0.407
DAO	0.316	0.875^**^	0.511	0.874^**^	0.947^**^	0.345	−	−0.532^*^	−0.432	0.953^**^	0.435	0.662^**^
H_2_O_2_	−0.472	−0.573^*^	−0.137	−0.572^*^	−0.762^**^	0.490	−0.532^*^	−	0.930^**^	−0.574^*^	−0.932^**^	−0.944^**^
MDA	−0.235	−0.493	−0.005	−0.554^*^	−0.678^**^	0.552^*^	−0.432	0.930^**^	−	−0.517^*^	−0.826^**^	−0.830^**^
SOD	0.197	0.935^**^	0.392	0.873^**^	0.946^**^	0.151	0.953^**^	−0.574^*^	−0.517^*^	−	0.530^*^	0.725^**^
POD	0.406	0.569^*^	0.109	0.441	0.666^**^	−0.600^*^	0.435	−0.932^**^	−0.826^**^	0.530^*^	−	0.921^**^
CAT	0.511	0.746^**^	0.161	0.679^**^	0.852^**^	−0.407	0.662^**^	−0.944^**^	−0.830^**^	0.725^**^	0.921^**^	−

Each square indicates the Pearson's correlation coefficient of a pair of parameters. Put, diamine putrescine (nmol/g^−1^ FW); Spd + Spm, triamine spermidine + tetraamine spermine (nmol g^−1^ FW); ADC, arginine decarboxylase (nmol(Agm)·g^−1^(FW)·h^−1^); ODC, ornithine decarboxylase (nmol(Put)·g^−1^(FW)·h^−1^); SAMDC, S-adenosylmethionine decarboxylase (nmol(SAM)·g^−1^(FW)·h^−1^); PAO, polyamine oxidase (U g^−1^ FW); DAO, diamine oxidase (U g^−1^ FW); H_2_O_2_, hydrogen peroxide (nmol g^−1^ FW); MDA, malondialdehyde (nmol g^−1^ FW); SOD, superoxide dismutase (U min^−1^ g^−1^ FW); POD, peroxidase (U min^−1^ g^−1^ FW); CAT, catalase (U min^−1^ g^−1^ FW). Correlations are significant at

* p < 0.05;

** *p < 0.01*.

**Table 5:** **Pearson's correlation coefficients among PA contents and physiological and biochemical parameters in cv. Z057 exposed to salt stress and treated with exogenous Spd (0, 0.15, 0.30, 0.45, 0.60 mM)**.

	**Put**	**Spd + Spm**	**ADC**	**ODC**	**SAMDC**	**PAO**	**DAO**	**H_2_O_2_**	**MDA**	**SOD**	**POD**	**CAT**
Put	−	−0.268	0.163	−0.586^*^	−0.303	0.475	0.308	0.422	0.043	−0.277	−0.104	0.173
Spd + Spm	−0.268	−	0.221	0.888^**^	0.903^**^	0.340	0.669^**^	−0.894^**^	−0.819^**^	0.956^**^	0.928^**^	0.839^**^
ADC	0.163	0.221	−	0.129	0.172	0.381	0.311	−0.071	−0.263	0.382	0.387	0.399
ODC	−0.586^*^	0.888^**^	0.129	−	0.886^**^	0.035	0.474	−0.900^**^	−0.726^**^	0.876^**^	0.761^**^	0.625^*^
SAMDC	−0.303	0.903^**^	0.172	0.886^**^	−	0.287	0.790^**^	−0.886^**^	−0.871^**^	0.922^**^	0.884^**^	0.849^**^
PAO	0.475	0.340	0.381	0.035	0.287	−	0.658^**^	−0.218	−0.405	0.290	0.448	0.595^*^
DAO	0.308	0.669^**^	0.311	0.474	0.790^**^	0.658^**^	−	−0.599^*^	−0.801^**^	0.700^**^	0.769^**^	0.925^**^
H_2_O_2_	0.422	−0.894^**^	−0.071	−0.900^**^	−0.886^**^	−0.218	−0.599^*^	−	0.818^**^	−0.815^**^	−0.814^**^	−0.682^**^
MDA	0.043	−0.819^**^	−0.263	−0.726^**^	−0.871^**^	−0.405	−0.801^**^	0.818^**^	−	−0.798^**^	−0.848^**^	−0.823^**^
SOD	−0.277	0.956^**^	0.382	0.876^**^	0.922^**^	0.290	0.700^**^	−0.815^**^	−0.798^**^	−	0.919^**^	0.860^**^
POD	−0.104	0.928^**^	0.387	0.761^**^	0.884^**^	0.448	0.769^**^	−0.814^**^	−0.848^**^	0.919^**^	−	0.885^**^
CAT	0.173	0.839^**^	0.399	0.625^*^	0.849^**^	0.595^*^	0.925^**^	−0.682^**^	−0.823^**^	0.860^*^	0.885^**^	−

Each square indicates the Pearson's correlation coefficient of a pair of parameters. Put, diamine putrescine (nmol/g^−1^ FW); Spd + Spm, triamine spermidine + tetraamine spermine (nmol g^−1^ FW); ADC, arginine decarboxylase (nmol(Agm)·g^−1^(FW)·h^−1^); ODC, ornithine decarboxylase (nmol(Put)·g^−1^(FW)·h^−1^); SAMDC, S-adenosylmethionine decarboxylase (nmol(SAM)·g^−1^(FW)·h^−1^); PAO, polyamine oxidase (U g^−1^ FW); DAO, diamine oxidase (U g^−1^ FW); H_2_O_2_, hydrogen peroxide (nmol g^−1^ FW); MDA, malondialdehyde (nmol g^−1^ FW); SOD, superoxide dismutase (U min^−1^ g^−1^ FW); POD, peroxidase (U min^−1^ g^−1^ FW); CAT, catalase (U min^−1^ g^−1^ FW). Correlations are significant at

* p < 0.05;

** *p < 0.01*.

## Discussion

In plants, salt stress causes reductions in fresh root weight, root length and relative root water contents, with severe damage to the organism, and in our study, such reductions were greater in cv. Z081 than in cv. Z057, indicating the salt tolerance of the latter (Table 2).

Exogenous Spd has been shown to act as a stimulant in a variety of organisms, and recent work indicates that exogenous Spd treatment enhances salt tolerance in plants (Liu et al., 2004). The plant PA metabolism response to salt stress varies with different exogenous Spd concentrations, plant species, and interactions among other stress factors (Gill and Tuteja, 2010). Indeed, our results showed different PA responses to salt stress in cv. Z081 and cv. Z057 in relation to different concentrations of exogenous Spd.

PA levels changed under salt stress, with Put decreasing and Spd and/or Spm increasing in most cases. It has been reported that with an increase in the (Spd + Spm)/Put ratio, salinity tolerance increased in all species examined (Zapata et al., 2004) and that Spd and Spm facilitate the osmotic stress tolerance of wheat seedlings (Liu et al., 2004). We investigated PA metabolism and the physiological responses of two cultivars of salt-stressed *Z. japonica* treated with different concentrations of exogenous Spd. Both cultivars first showed an upward trend followed by a downward trend in total Spd and Spm contents under salt stress and the significant changes in the three forms of PAs under different treatment with concentrations of exogenous Spd suggest an efficient PA adaptive mechanism (Figure 2). Exogenous Spd inhibited accumulation of free Put and promoted accumulation of free Spd and Spm as well as both soluble conjugated and insoluble bound Put, Spd, and Spm (Figure 2). The importance of soluble conjugated and insoluble bound PAs has also been illustrated in previous work, and overexpression of the SAMDC gene in tobacco significantly increased the concentrations of soluble conjugated PAs (Jia et al., 2010).

Expression of several genes encoding enzymes of PA metabolism, such as ADC, ODC, or SAMDC, were found to improve environmental stress tolerance in most plant species, revealing a useful tool for gaining new insight into the regulation of PA metabolism (Bagni and Tassoni, 2001; Liu et al., 2007). Synthesis of the diamine Put proceeds through either ADC via agmatine (Agm) or ODC, whereas the triamine Spd is synthesized by SPDS via addition to Put of an aminopropyl moiety donated by decarboxylated S-adenosylmethionine (dcSAM) formed by SAMDC (Franceschetti et al., 2004). The results of the present study show a significant positive correlation between the Spd + Spm content and the activities of ADC and SAMDC with exogenous Spd application in both cultivars, proving that such variation in exogenous Spd concentration affected PA metabolism by altering the activity of ADC and SAMDC (Figure 3, Tables 3, 4). Previous studies have reported that exogenous Put is quickly absorbed and converted to Spd and Spm and that synthetic PAs accumulate in stems and roots and alter endogenous PA contents (Ohe et al., 2005).

PAs are catabolized into ammonia and H_2_O_2_ by DAO and PAO (Figure 1). These enzymes are localized in the plant cell wall, and hydrogen peroxide resulting from Put catabolism may be important in cross-linking reactions under both normal and stress conditions (Eller et al., 2006). In both cultivars, we observed a possible connection between the PA content and degradation enzyme with different exogenous Spd concentrations. Our results showed a positive correlation between increases in PA contents and DAO in both cultivars, which indicated that the increase in PA contents was due to the activity of DAO rather than PAO (Figure 4).

Salt stress leads to the generation of reactive oxygen species, such as H_2_O_2_, which cause lipid peroxidation and disturb normal cellular metabolism. PAs also reversed salinity-induced reductions in seedling growth and biomass accumulation and increased ${\text{O}}_{2}^{-}$-, H_2_O_2_, and MDA levels and the activity of antioxidant enzymes and carotenoids in salt-stressed *Brassica juncea* seedlings (Verma and Mishra, 2005). Our study shows that as exogenous Spd increased, the concentrations of H_2_O_2_ and MDA were augmented and then diminished in the roots of both cultivars, and this defense response was most likely due to the increase in antioxidant enzyme activity (Matsuda, 1984). Our research showed that SOD, POD, and CAT activities increased significantly in salt-stressed roots when treated with different concentrations of exogenous Spd (Figure 6). With changing exogenous Spd concentration, we found a positive correlation between the Spd + Spm content and antioxidant enzyme activities but a negative correlation between the Spd + Spm content and H_2_O_2_ and MDA levels in response to salt stress (Figure 5).

In summary, although exhibiting different responses, the two zoysiagrass cultivars shared similar PA metabolism and physiological and biochemical mechanisms in response to salt stress with increasing exogenous Spd concentration. By enhancing the activities of ODC, DAO, and SAMDC, cv. Z057 showed a better salt stress adaptation ability with exogenous Spd application, which promoted the conversion of Put into Spd and Spm. The addition of exogenous Spd further induced antioxidant enzyme activities, reduced H_2_O_2_ and MDA levels, and improved the tolerance of zoysiagrass to salinity stress.

Although our findings are important for academic research and cultivation, more research is needed to determine the number and frequency of exogenous Spd applications required to achieve optimal zoysiagrass growth.

## Author contributions

SL designed research; SL performed research; HJ contributed new reagents/analytic tools; SL and HJ analyzed data; and SL, HJ, and QZ wrote the paper.

### Conflict of interest statement

The authors declare that the research was conducted in the absence of any commercial or financial relationships that could be construed as a potential conflict of interest.
